# Development and content validation of an interprofessional collaboration model for child sexual abuse prevention in primary health care

**DOI:** 10.1186/s12875-026-03352-z

**Published:** 2026-05-12

**Authors:** Flora Niu, Meita Dhamayanti, Elsa Pudji Setiawati, Nita Arisanti

**Affiliations:** 1 Doctoral Program in Medical Sciences, Faculty of Medicine, Universitas Padjadjaran, Bandung, West Java Indonesia; 2 Department of Child Health, Hasan Sadikin Hospital, Faculty of Medicine, Universitas Padjadjaran, Bandung, West Java Indonesia; 3 Department of Public Health, Universitas Padjadjaran, Bandung, West Java Indonesia; 4 Department of Public Health, Faculty of Medicine, Universitas Padjadjaran, Bandung, West Java Indonesia

**Keywords:** Child sexual abuse, Interprofessional collaboration, Primary health care, Content validity, Child protection

## Abstract

**Background:**

Child sexual abuse (CSA) remains a significant public health issue requiring effective prevention strategies within primary health care (PHC). Interprofessional collaboration (IPC) is recognized as a key approach; however, context-specific IPC models for CSA prevention are still limited, particularly in low- and middle-income settings.

**Objective:**

This study aimed to develop and conduct content validation of an interprofessional collaboration model for CSA prevention in primary health care settings.

**Methods:**

The model was developed through a structured narrative literature review and conceptual synthesis, followed by expert evaluation. Seven experts from diverse professional backgrounds assessed the relevance of the model indicators using the Content Validity Index (CVI). Indicators were refined based on qualitative feedback to improve clarity and measurability.

**Results:**

The final model comprised 27 indicators across seven dimensions of IPC. All indicators demonstrated high item-level content validity (I-CVI = 1.00), indicating strong agreement among experts. The scale-level content validity (S-CVI/Ave) also indicated excellent overall content validity.

**Conclusion:**

This study provides preliminary evidence of content validity for an IPC model to support CSA prevention in primary health care settings. The findings reflect expert agreement on indicator relevance rather than empirical validation or real-world effectiveness. Further research is required to evaluate construct validity, reliability, and feasibility in practice.

## Introduction

Child sexual abuse (CSA) is a major global public health issue with long-term physical, psychological, and social consequences. These impacts highlight the urgent need to shift from reactive responses toward preventive strategies [1–3]. 

Primary health care (PHC) plays a critical role in CSA prevention due to its accessibility, continuity of care, and early contact with children and families. These characteristics enable early identification of risk factors, provision of preventive education, and coordination of interprofessional responses. Furthermore, the longitudinal nature of PHC services allows continuous monitoring and follow-up, which are essential for strengthening preventive interventions and ensuring child safety [3–5]. 

Evidence from systematic reviews indicates that effective interprofessional collaboration (IPC) improves communication, role clarity, shared decision-making, and coordination of services within primary health care teams. These collaborative processes are particularly relevant in addressing complex child protection issues such as CSA, where coordinated actions among health professionals, social workers, psychologists, and child protection agencies are required [6, 7]. 

Despite the recognized benefits of IPC in improving health service delivery, the implementation of collaborative practices within PHC settings remains inconsistent and often depends on organizational structures, leadership support, and communication mechanisms within teams [8–12]. However, most existing IPC models have been developed for chronic disease management or general primary care outcomes and do not specifically address the complexities of child sexual abuse (CSA) prevention in primary health care settings.

Few studies provide validated and operational IPC models specifically designed for CSA prevention in PHC settings, particularly in low- and middle-income countries [5, 13–15]. This gap suggests the need for a structured framework that integrates IPC principles with child protection strategies within PHC systems. In addition, there is a lack of context-specific and content-validated IPC frameworks designed to support CSA prevention in low- and middle-income countries, including Indonesia. Therefore, this study aims to address this gap by developing and conducting content validation of an IPC model specifically tailored to strengthen CSA prevention in primary health care settings.

The importance of contextualizing such models is particularly relevant in low- and middle-income countries, including Indonesia, where primary health care is largely delivered through community-based health centers (Puskesmas) that serve as the cornerstone of preventive and promotive health services. Within this system, health professionals frequently interact with children and families through routine maternal and child health programs, immunization services, and community outreach activities. These interactions provide important opportunities to strengthen preventive strategies for child protection. However, coordination between health services and other sectors involved in child protection often remains limited, which may hinder effective preventive action [16–18]. 

In Indonesia, CSA remains a significant concern, with national data from the Indonesian Child Protection Commission (KPAI) reporting over 2,000–2,700 cases annually. However, these figures likely underestimate the true burden due to underreporting influenced by stigma and socio-cultural barriers [19, 20]. 

Although multiple sectors are involved in child protection, collaboration among professionals within primary health care services remains insufficiently structured, potentially limiting the effectiveness of early preventive actions [21, 22]. 

While early detection and response are important elements of child protection, prevention remains a fundamental strategy for reducing the occurrence and long-term impact of CSA [23]. Strengthening collaborative capacity among PHC professionals can enhance awareness of risk factors, improve communication among service providers, and facilitate coordinated preventive efforts before abuse occurs or escalates [24]. In this context, the development of a structured interprofessional collaboration model focused on prevention may support more effective coordination within PHC teams and improve the integration of child protection principles into routine health services [25, 26]. 

Although detection and response are important components of child protection, this study specifically focuses on primary prevention, aiming to strengthen early collaborative efforts to reduce the risk of CSA before it occurs [27]. 

Therefore, this study aimed to develop and conduct content validation of an interprofessional collaboration model specifically designed to strengthen the prevention of child sexual abuse within primary health care settings using an expert judgment approach. By integrating evidence from international literature with expert consensus and contextual considerations relevant to PHC systems, this study seeks to provide a theoretically grounded and practically applicable framework to guide collaborative preventive practices and future research in child protection within primary health care.

**Fig. 1:**
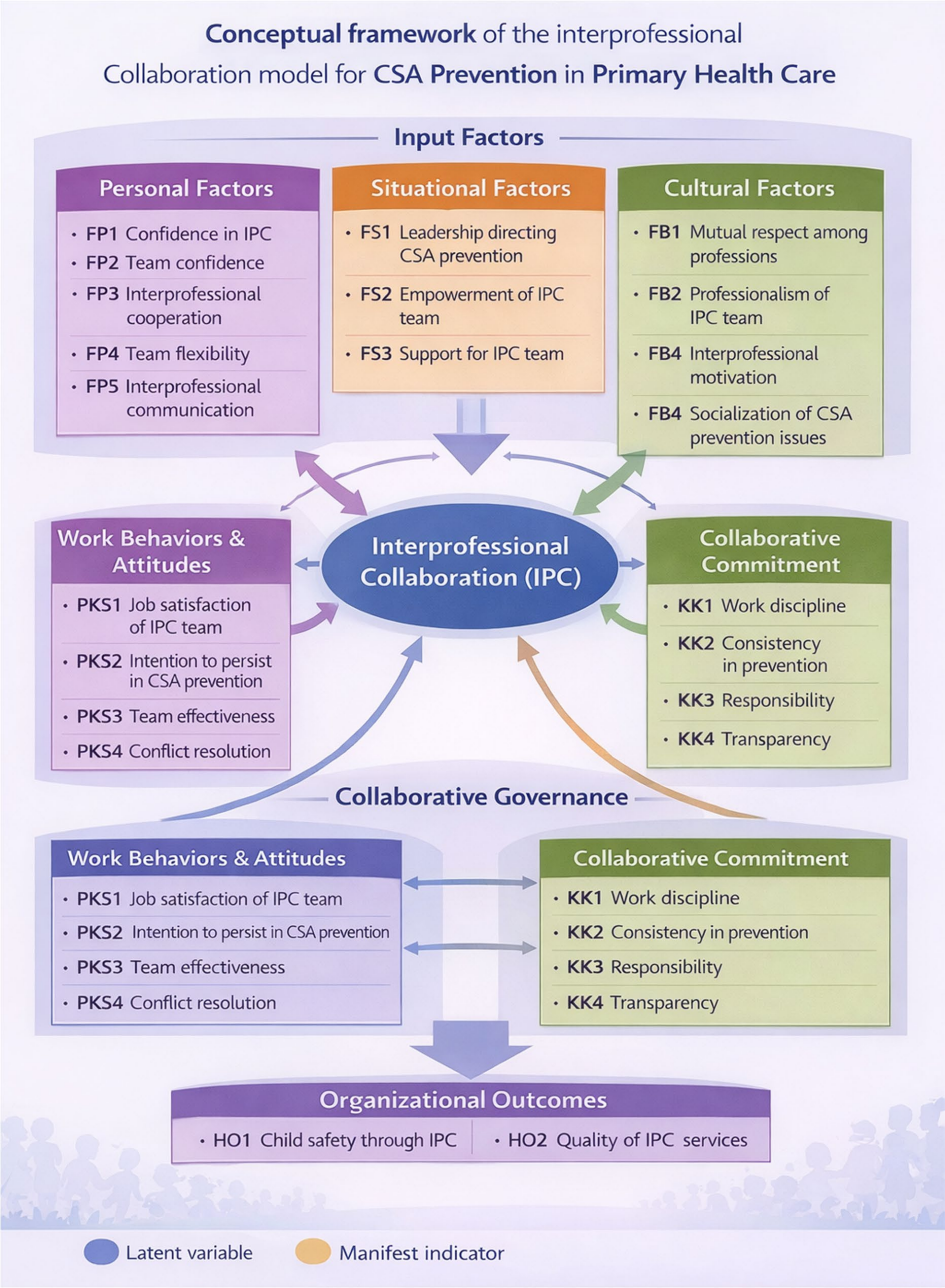
Conceptual framework of interprofessional collaboration (IPC) for child sexual abuse (CSA) prevention in primary health care

To understand the relationships among the variables in this study, a conceptual framework of the interprofessional collaboration (IPC) model for the prevention of child sexual abuse (CSA) in primary health care was developed. The framework integrates personal factors, situational factors, and cultural factors as input components influencing IPC implementation. Furthermore, work behaviors and attitudes, collaborative commitment, and collaborative governance strengthen the IPC process and lead to organizational outcomes, including improved child safety and quality of collaborative services. Figure 1 is an original conceptual framework developed by the authors based on literature synthesis. No copyrighted material was used or adapted in this figure. Figure 1. Conceptual framework of the interprofessional collaboration (IPC) model for the prevention of child sexual abuse (CSA) in primary health care, illustrating the relationships between input factors, collaborative processes, and organizational outcomes.

## Methods

### Study design

This study employed a multi-phase model development and content validation design consisting of three sequential stages: (1) development of an initial conceptual model based on literature synthesis, (2) expert panel evaluation using a structured assessment process, and (3) quantitative content validity assessment using the Content Validity Index (CVI) [27, 28]. 

This approach is widely used in health systems research to ensure that conceptual models adequately represent relevant theoretical constructs before further empirical testing [25, 27]. 

This study adhered to the ethical principles outlined in the Declaration of Helsinki. The human participants consisted of professional experts involved in an expert evaluation process. All participants received detailed information regarding the study’s objectives, procedures, and their rights before participating. Written informed consent was obtained from all participants. Participation was completely voluntary, and participants had the right to withdraw at any time without consequence. To ensure confidentiality, all responses were anonymized, and no identifiable information was disclosed.

Ethics approval for this study was obtained from the Research Ethics Committee of Padjadjaran University (Approval Number: 234/UN6.KEP/EC/2025). This study involved expert evaluation and did not include patient data or clinical interventions.

### Development of the initial model

The initial conceptual model of interprofessional collaboration for CSA prevention in PHC was developed through a structured narrative literature review. The review aimed to identify key components of IPC frameworks, child protection systems, and collaborative practices relevant to CSA prevention in primary health care settings. A narrative review approach was used to allow flexible integration of diverse conceptual frameworks relevant to IPC and CSA prevention [7, 27, 29].

### Literature search strategy

A comprehensive literature search was conducted in PubMed, Scopus, Web of Science, and Google Scholar covering publications from January 2000 to January 2026 [30]. The final search was performed on January 10, 2026, using combinations of the following keywords: interprofessional collaboration, primary health care, child protection, child sexual abuse prevention and collaborative practice. Boolean operators (AND/OR) were used to refine the search strategy [29, 31]. A PRISMA flow diagram illustrating the study selection process is provided in Supplementary Fig. 1.

### Inclusion criteria

Studies were included if they: discussed interprofessional collaboration in health care or primary care settings, addressed child protection, child maltreatment, or CSA prevention described collaborative frameworks, teamwork models, or organizational mechanisms relevant to IPC, and were published in peer-reviewed journals in English. A total of 40 records were identified through database searches and additional sources. After removing duplicates (*n* = 20), 20 records remained for screening. Following title and abstract screening, 10 records were excluded. The remaining 10 full-text articles were assessed for eligibility, with all meeting the inclusion criteria and included in the final analysis [31, 32]. A PRISMA flow diagram illustrating the study selection process is provided in Supplementary Fig. 1.

### Data extraction and synthesis

Data extraction involved identifying key concepts related to interprofessional collaboration and child sexual abuse prevention from the included studies [24, 33–35]. These concepts were treated as meaningful units and subjected to open coding, with each concept assigned a descriptive label representing underlying principles [27, 36, 37]. Codes with similar meanings were grouped into categories, which were then synthesized into broader themes through an iterative process of comparison and refinement. The resulting themes were then organized into seven overarching dimensions representing core components of interprofessional collaboration in primary care settings. Each dimension was then operationalized into measurable indicators by translating abstract concepts into observable elements, resulting in a total of 27 indicators.

Conceptually overlapping indicators were systematically reviewed and refined to improve clarity and reduce redundancy. The refinement process, including merging, differentiation, and rewording of indicators, is presented in Table 1.

**Table 1:** Refinement of Conceptually Overlapping Indicators in the IPC Model

No	Conceptual Domain	Original Indicators	Identified Issue	Refinement Strategy	Final Indicator
1	Team Commitment	Work discipline, Consistency, Responsibility	High redundancy (all reflect adherence and commitment)	Merged into a single construct	Consistency in implementing IPC practices for CSA prevention
2	Interprofessional Interaction	Collaboration, Communication	Conceptual overlap (communication embedded within collaboration)	Differentiated into distinct constructs	Clear and structured communication; Joint decision-making and collaboration
3	Psychological Factors	Trust, Team efficacy (belief in IPC)	Overlapping psychological constructs	Conceptually distinguished	Confidence in team performance; Mutual trust among professionals
4	Leadership & Support	Leadership, Empowerment, Support	Overlapping leadership-related constructs	Reframed as complementary components	Leadership facilitating collaboration; Empowerment of team members; Organizational support
5	Professional Culture	Mutual respect, Professionalism, Motivation	Overlap in professional values	Differentiated	Respect for professional roles; Professionalism; Motivation for collaboration
6	Organizational System	Team structure, Coordination	Functional overlap	Separated	Clear team structure; Coordination of interprofessional tasks
7	Outcome Measures	Team effectiveness, Service quality, Patient safety	Overlap in outcomes	Separated by level	Team effectiveness; Quality of IPC services; Patient safety
8	Communication Transparency	Transparency, Communication	Partial overlap	Clarified	Transparency in interprofessional communication
9	Adaptability	Flexibility	Too broad	Operationalized	Ability to adapt roles and responsibilities in IPC practice
10	Conflict Management	Conflict handling	Not specific	Clarified	Management of interprofessional conflicts
11	Knowledge Dissemination	Socialization of CSA prevention	Not measurable	Removed / integrated	Integrated into training and communication processes

During the model development process, the initial set of indicators was systematically reviewed to identify potential conceptual overlap. This step aimed to ensure that each indicator represented a distinct construct and to minimize redundancy.

Indicators with similar meanings or overlapping conceptual domains were refined through a structured process involving merging redundant items, differentiating closely related constructs, and rewording indicators to enhance clarity and operational definition. This refinement process was conducted iteratively through discussion among the research team to ensure conceptual consistency and alignment with the study objectives [27, 36, 37]. 

In addition to addressing conceptual overlap, several indicators were identified as vague or insufficiently operationalized. These indicators were refined to improve clarity, specificity, and measurability. Abstract or general terms such as ‘flexibility’, ‘motivation’, and ‘transparency’ were reworded into more concrete and observable forms to ensure that each indicator could be reliably assessed [38–40]. This refinement process involved transforming broad concepts into measurable indicators by specifying behaviors, actions, or observable outcomes relevant to interprofessional collaboration in CSA prevention [27, 28, 41, 42]. All revisions were conducted iteratively through discussion among the research team to ensure conceptual clarity and alignment with the study objectives.

This process was conducted iteratively with ongoing discussions among the research team to ensure conceptual clarity, consistency, and contextual relevance. Through this process, seven conceptual dimensions were identified: personal factors, situational factors, work behaviors and attitudes, organizational outcomes, collaborative commitment, cultural factors, and collaborative governance [27, 28, 41, 42]. These dimensions were operationalized into 27 preliminary indicators, which formed the initial IPC model, which was then validated by experts.

The qualitative findings referred to in this study were derived from thematic synthesis of the included literature rather than primary qualitative data collection.

### Expert panel selection

Experts were identified through professional networks and selected based on predefined eligibility criteria. Invitations were sent via email, and participation was voluntary. Eligible experts were required to have relevant experience in primary health care, interprofessional collaboration, or child protection, and a minimum of 10 years of professional or academic experience [43–46]. 

Experts were recruited through purposive sampling using professional networks and formal invitations via email. The number of experts (*n* = 7) was considered adequate for content validity studies, as recommended in methodological literature (5–10 experts) [43, 46, 47]. 

### Eligibility criteria for experts

Experts were eligible if they met the following criteria: minimum of 10 years of professional experience in relevant fields, active involvement in clinical practice, academic research, policy development, or professional training related to child protection or primary health care, and demonstrated knowledge or experience in interprofessional collaboration or child maltreatment prevention.

### Composition of the expert panel

Seven experts participated in the validation process. The panel represented multiple disciplines relevant to the study topic, including: pediatrics and child health, child protection services, primary health care systems, interprofessional collaboration and workforce development, child psychology and interprofessional education.

This multidisciplinary composition ensured that the model indicators were evaluated from clinical, psychosocial, organizational, and policy perspectives [38, 48]. 

### Conflict of interest

Experts were independent professionals who were not directly involved in the development of the model. Prior to participation, all experts were informed about the purpose of the study and confirmed that they had no conflicts of interest related to the evaluation process.

### Content validity

The initial model consisting of seven dimensions and 27 indicators was distributed to the expert panel for evaluation. Experts were asked to assess each indicator using a four-point ordinal rating scale based on its relevance to interprofessional collaboration for CSA prevention in PHC [27, 39, 43]. 

The rating scale consisted of:

1. not relevant
2. somewhat relevant
3. quite relevant
4. highly relevant

Experts assessed both the relevance and clarity of each indicator, although the CVI calculation was based on relevance ratings. Experts were also invited to provide qualitative comments and suggestions regarding the clarity, wording, and contextual relevance of each indicator. Experts independently rated each indicator in a single-round assessment; no Delphi rounds were conducted. In addition to quantitative ratings, qualitative feedback provided by the experts was systematically reviewed and analyzed. Comments were categorized based on common themes, such as wording clarity, relevance, and contextual appropriateness. Decisions to revise, retain, or remove items were made through discussion and consensus among the research team, taking into account both the CVI scores and the qualitative feedback from experts. Items with low relevance scores or unclear wording were revised to improve clarity and alignment with the intended construct [39, 40, 43]. 

Where discrepancies in expert opinions occurred, the research team prioritized suggestions that improved conceptual clarity and consistency with the overall model framework.

### Data analysis

Qualitative and quantitative analyses were conducted sequentially to ensure integration of expert feedback into model refinement.

### Quantitative analysis

Content validity was evaluated using the Content Validity Index (CVI). Item-Level Content Validity Index (I-CVI) The I-CVI represents the proportion of experts who rated each item as relevant (score of 3 or 4) [27, 39, 43]. It was calculated using the following formula:

$$\mathrm{I-CVI=}\frac{\text{Number of experts rating item as relevant}}{\text{Total number of experts}}$$

For a panel of seven experts, an I-CVI threshold of ≥ 0.78 was considered acceptable [27, 39]. 

Scale-level content validity was calculated using the average method (S-CVI/Ave), obtained by averaging the I-CVI scores across all indicators within each dimension [27, 39]. 

Items falling below the recommended threshold were reviewed for potential revision or removal.

Qualitative and quantitative analyses were conducted sequentially to ensure integration of expert feedback into model refinement [43, 46]. 

Modified kappa statistics were not calculated in this study. Although kappa can provide additional adjustment for chance agreement, the Content Validity Index (CVI) remains a widely accepted and commonly used method for assessing content validity in early-stage instrument and model development studies, particularly with small expert panels. Given the exploratory nature of this study and the relatively small number of experts, CVI was considered sufficient to establish initial content validity. Future studies are recommended to incorporate modified kappa statistics to further strengthen the robustness of the validation process [25, 27]. 

### Qualitative feedback analysis

In addition to the quantitative CVI assessment, qualitative feedback provided by experts was systematically reviewed. Qualitative feedback was analyzed using thematic categorization. Comments from experts were independently reviewed, coded, and grouped into themes [24, 33–35, 49]. This process ensured that revisions were systematically incorporated into the model. Comments were categorized according to themes such as: clarity of wording, conceptual relevance, contextual applicability in PHC settings and suggested modifications.

These comments informed minor revisions to indicator wording to enhance clarity and ensure consistency with primary health care practice [33–35, 49]. 

## Results

### Expert panel characteristics

The expert panel consisted of seven professionals with extensive experience in relevant fields, including child health, child protection, interprofessional collaboration, psychology, and primary health care systems. All experts had substantial professional experience ranging from 15 to over 30 years, ensuring a high level of expertise in their respective fields.

The panel represented diverse institutional affiliations and practice settings, including clinical practice (pediatricians and primary health care practitioners), academic and training institutions (interprofessional education experts), and child protection services. This diversity ensured the inclusion of perspectives from health service delivery, education, and system-level implementation.

The experts engaged in diverse roles such as clinical care, psychosocial assessment, workforce development, and policy-relevant child protection practices, contributing to a comprehensive evaluation of the model from a variety of professional and contextual perspectives.

The experts came from diverse geographic regions, ensuring a diverse representation of contextual experiences in primary care.

This composition supported a comprehensive evaluation of the model from clinical, psychosocial, organizational, and health system perspectives. Detailed characteristics of the expert panel are presented in Table 2.

**Table 2:** Characteristics of the Expert Panel (*n* = 7)

No	Area of Expertise	Professional Background	Years of Experience	Institutional Affiliation	Practice Setting	Field of Contribution to the Study
1	Child Health / Pediatrics	Pediatrician / Child Health Specialist	≥ 20 years	Hospital / Academic Medical Center	Clinical (Secondary & Primary Care)	Clinical perspectives on child health, early identification of CSA, and preventive care in PHC
2	Child Protection Services	Child Protection Practitioner	≥ 20 years	Government / Child Protection Agency	Community & Social Services	Practical experience in child protection systems, referral pathways, and intersectoral collaboration
3	Interprofessional Collaboration	Health Workforce / IPC Expert	≥ 20 years	University / Research Institution	Academic & Training	Theoretical and practical expertise in IPC models, teamwork, and collaborative practice
4	Child Psychology	Clinical / Child Psychologist	≥ 15 years	Hospital / Private Practice / Academic Institution	Clinical & Psychosocial Services	Psychosocial assessment, trauma-informed care, and child-centered preventive approaches
5	Primary Health Care Systems	Primary Health Care Practitioner	16 years	Primary Health Care Center (PHC)	Primary Care	Population-based prevention strategies and primary health care system strengthening
6	Child Protection in PHC	Primary Care Child Protection Specialist	≥ 20 years	Primary Health Care Center / Public Health Office	Primary Care & Public Health	Integration of child protection principles into PHC services and referral pathways
7	Interprofessional Education	IPE Trainer for Primary Care Teams	31 years	University / Training Institution	Academic & Capacity Building	Capacity building and competency development for IPC in primary health care teams

### Structure of the model

The initial IPC model submitted for validation consisted of seven conceptual dimensions and 27 indicators derived from the literature review and theoretical synthesis. Each indicator reflects specific observable or organizational aspects of IPC implementation in CSA prevention.

The seven dimensions included personal factors, situational factors, work behaviors and attitudes, collaborative commitment, cultural factors, collaborative governance, and organizational outcomes:

1. Personal Factors: Confidence of IPC team in CSA prevention, Interprofessional collaboration in CSA prevention, flexibility of IPC team in CSA prevention and Interprofessional communication in CSA prevention.
2. Situational Factors: Leadership in directing to prevent CSA, empowerment of IPC Team to prevent CSA and support of IPC team in preventing CSA.
3. Work Behavior and Attitude: Job satisfaction among IPC team members in CSA prevention, IPC team intention to persist in preventing CSA, team effectiveness in preventing CSA and IPC team conflict resolution in preventing CSA.
4. Organizational Outcomes: CSA patient safety through the IPC Team and the quality of IPC Team services in preventing CSA.
5. Collaborative Commitment: IPC Team’s work discipline in preventing CSA, IPC Team’s consistency in preventing CSA, IPC Team’s responsibility in preventing CSA and IPC Team’s transparency in preventing CSA.
6. Cultural Factors: Mutual Respect between professions in preventing CSA, Professionalism of the IPC Team in preventing CSA, Motivation between professions in preventing CSA and Socialization of CSA prevention issues.
7. Collaborative Governance: SOP in preventing CSA, Coordination between professions in preventing CSA, Strengthening HR in preventing CSA, Team work structure in preventing CSA and Cost support in preventing CSA.

Each dimension contained between 2 and 5 indicators, resulting in a total of 27 indicators evaluated by the expert panel.

Several indicators were revised to enhance clarity and ensure they could be operationalized into measurable components, thereby improving the precision of the model.

### Dimensions and indicators

The development of the interprofessional collaboration (IPC) model for the prevention of child sexual abuse (CSA) was based on the integration of qualitative findings and a review of relevant literature on collaborative practice in primary health care. The qualitative analysis identified key themes related to factors that influence effective collaboration among health professionals in preventing CSA. These themes were subsequently translated into measurable dimensions and indicators to operationalize the IPC model. In total, the model consists of seven dimensions and 27 indicators, representing personal factors, situational factors, work behaviors and attitudes, organizational outcomes, collaborative commitment, cultural factors, and collaborative governance. These indicators reflect the essential components required to support effective interprofessional collaboration in CSA prevention within primary health care settings. The detailed operationalization of each dimension and indicator is presented in Table 3.

**Table 3:** Dimensions and Indicators of the Interprofessional Collaboration Model for Child Sexual Abuse Prevention

Dimension	Indicator Code	Indicator Description
Personal Factors	PF1	Members of the IPC team have confidence in the collective capability of the team to implement CSA prevention efforts.
	PF2	Health professionals demonstrate confidence in participating in interprofessional collaboration (IPC) activities aimed at preventing child sexual abuse (CSA).
	PF3	Health professionals actively engage in collaborative practices with other disciplines to support CSA prevention.
	PF4	IPC team members demonstrate flexibility and adaptability when working collaboratively to address CSA prevention challenges.
	PF5	Effective interprofessional communication occurs among team members when addressing CSA prevention issues.
Situational Factors	SF1	Leadership within the health care organization provides clear direction and guidance for CSA prevention through collaborative practice.
	SF2	Health professionals are empowered to participate actively in IPC activities related to CSA prevention.
	SF3	Organizational and managerial support facilitates collaborative teamwork for CSA prevention within primary health care settings.
Work Behaviors and Attitudes	WB1	Members of the IPC team experience job satisfaction when participating in collaborative efforts to prevent CSA.
	WB2	IPC team members demonstrate a strong intention to continue participating in collaborative CSA prevention activities.
	WB3	The IPC team demonstrates effectiveness in performing collaborative tasks aimed at preventing CSA.
	WB4	Team members are able to resolve interprofessional conflicts constructively when addressing CSA prevention cases.
Organizational Outcomes	OO1	Interprofessional collaboration contributes to improved patient safety for children at risk of CSA.
	OO2	Collaborative practice enhances the overall quality of services provided by the IPC team in preventing CSA.
Collaborative Commitment	CC1	IPC team members demonstrate work discipline in carrying out collaborative responsibilities related to CSA prevention.
	CC2	Professionals maintain consistency in implementing collaborative actions for CSA prevention.
	CC3	IPC team members demonstrate a strong sense of responsibility in protecting children through collaborative practice.
	CC4	Transparency is maintained among IPC team members when sharing information and making decisions related to CSA prevention.
Cultural Factors	CF1	Mutual respect is demonstrated among professionals from different disciplines involved in CSA prevention
	CF2	IPC team members demonstrate professionalism when collaborating to address CSA prevention issues.
	CF3	Professionals motivate and support one another in collaborative efforts aimed at preventing CSA.
	CF4	CSA prevention issues are actively socialized and discussed among professionals within the organization.
Collaborative Governance	CG1	Standard operating procedures (SOPs) are established to guide collaborative practices for CSA prevention.
	CG2	Effective coordination mechanisms exist between professionals involved in CSA prevention.
	CG3	Human resource capacity is strengthened to support collaborative CSA prevention activities.
	CG4	A clear teamwork structure supports coordinated interprofessional collaboration for CSA prevention
	CG5	Adequate financial or resource support is available to sustain collaborative CSA prevention programs.

### Content validity

As shown in Table 4, all 27 indicators achieved an item-level content validity index (I-CVI) value of 1.00, indicating that all seven experts rated each item as relevant. This reflects perfect agreement among experts regarding the relevance of the indicators included in the model. The consistent I-CVI value of 1.00 across all items suggests strong content validity, demonstrating that each indicator is considered highly relevant and appropriate for representing the construct of interprofessional collaboration in the context of child sexual abuse prevention.

**Table 4:** Item-Level Content Validity Index (I-CVI) for IPC Model Indicators

No	Item	Experts in Agreement (*n* = 7)	I-CVI	Interpretation
1	Q1	7	1.00	Strong content validity
2	Q2	7	1.00	Strong content validity
3	Q3	7	1.00	Strong content validity
4	Q4	7	1.00	Strong content validity
5	Q5	7	1.00	Strong content validity
6	Q6	7	1.00	Strong content validity
7	Q7	7	1.00	Strong content validity
8	Q8	7	1.00	Strong content validity
9	Q9	7	1.00	Strong content validity
10	Q10	7	1.00	Strong content validity
11	Q11	7	1.00	Strong content validity
12	Q12	7	1.00	Strong content validity
13	Q13	7	1.00	Strong content validity
14	Q14	7	1.00	Strong content validity
15	Q15	7	1.00	Strong content validity
16	Q16	7	1.00	Strong content validity
17	Q17	7	1.00	Strong content validity
18	Q18	7	1.00	Strong content validity
19	Q19	7	1.00	Strong content validity
20	Q20	7	1.00	Strong content validity
21	Q21	7	1.00	Strong content validity
22	Q22	7	1.00	Strong content validity
23	Q23	7	1.00	Strong content validity
24	Q24	7	1.00	Strong content validity
25	Q25	7	1.00	Strong content validity
26	Q26	7	1.00	Strong content validity
27	Q27	7	1.00	Strong content validity

No indicators demonstrated borderline validity (I-CVI = 0.83) or low validity, indicating that all items met and exceeded the recommended threshold for content validity. These findings indicate a high level of consensus among experts and suggest that the developed indicators are conceptually clear, well-defined, and aligned with the intended domains of the model.

Table 4. presents the item-level content validity index (I-CVI) for all indicators, demonstrating the level of agreement among experts regarding the relevance of each item.

### Qualitative feedback

In addition to the quantitative ratings, experts provided qualitative feedback on the proposed indicators. The comments were analyzed and categorized into several themes:

1. Clarification of Indicator Wording.

Some experts suggested minor modifications to improve the clarity and precision of several indicators, particularly those related to professional competencies and collaborative responsibilities.

(2) Alignment with Primary Health Care Context.

Several experts recommended refining terminology to better reflect the operational context of PHC services, including the roles of frontline health professionals and coordination with child protection systems.

(3) Emphasis on Governance and Leadership.

Experts highlighted the importance of leadership commitment, institutional policies, and clear referral mechanisms in supporting sustainable collaboration within PHC teams.

(4) Cultural Sensitivity in Collaborative Practice.

Feedback emphasized the need to acknowledge cultural and community contexts that may influence communication, trust, and collaborative decision-making among professionals.

Based on this feedback, minor wording revisions were made to several indicators to enhance clarity and contextual relevance, while the conceptual structure of the model remained unchanged.

Qualitative feedback from experts was systematically analyzed and used to refine indicator wording, improve clarity, and enhance contextual relevance.

Based on these thematic categories, qualitative feedback was systematically translated into specific revisions of the model indicators. Each suggested modification was reviewed and incorporated to improve clarity, contextual relevance, and operational definition, while preserving the conceptual integrity of the model.

Table 5 provides illustrative examples of how expert feedback informed the refinement of selected indicators. These examples reflect the key themes identified in the qualitative analysis, including clarification of wording, improvement of measurability, contextual adaptation to primary health care settings, and enhancement of conceptual precision.

**Table 5:** Examples of Indicator Revisions Based on Expert Feedback

No	Original Indicator	Expert Feedback	Revision Made	Final Indicator
1	Flexibility	Too vague	Clarified wording	Ability to adapt roles in IPC
2	Motivation	Not measurable	Made specific	Motivation to engage in IPC
3	Transparency	Unclear context	Added context	Transparency in interprofessional communication
4	Conflict	Too general	Operationalized	Management of interprofessional conflicts

### Final model

Following the content validation process and minor refinements based on expert feedback, the IPC model retained all seven dimensions and 27 indicators.

The validated model integrates individual, interpersonal, organizational, cultural, and governance components that collectively support collaborative practice in PHC settings for CSA prevention. The high level of agreement observed across both quantitative and qualitative evaluations indicates that the model provides a conceptually coherent and contextually appropriate framework for strengthening interprofessional collaboration in child protection within primary health care.

## Discussion

### Interpretation of the validated IPC model

The findings of this study demonstrate a high level of expert agreement regarding the relevance of the proposed indicators, as reflected by the uniformly high I-CVI values. However, these results should be interpreted as evidence of content validity rather than empirical effectiveness. The model has not yet been tested in real-world settings, and further studies are required to evaluate its construct validity, reliability, and practical applicability [27, 39, 43]. The results of this study represent an initial step in model development, focusing on content validation rather than empirical testing.

The conceptual model developed in this study integrates determinants, processes, and outcomes within a coherent framework of interprofessional collaboration. Determinants, such as personal and situational factors, are proposed to influence interprofessional collaboration processes, including communication, coordination, and team functioning. These collaborative processes, in turn, are expected to contribute to improved outcomes, such as team effectiveness, service quality, and patient safety in the context of child sexual abuse prevention. This structure reflects a systems-based perspective, where contextual and individual factors shape team processes, which subsequently determine organizational and service outcomes [8–11, 33–35]. 

This study developed and validated an interprofessional collaboration (IPC) model aimed at strengthening the prevention of child sexual abuse (CSA) in primary health care (PHC). The findings indicate that the proposed model achieved a high level of expert agreement across seven dimensions and 27 indicators. The CVI results suggest that the model components are generally considered relevant for supporting collaborative practice in CSA prevention within PHC settings. This finding aligns with recent studies highlighting the importance of interprofessional collaboration in preventive child protection strategies [3, 4, 8, 9, 17, 30]. 

Compared to previous IPC models developed in high-income settings, this model emphasizes preventive collaboration in resource-constrained PHC systems, highlighting its contextual relevance. This distinction is particularly important in low- and middle-income countries where health systems face structural and resource limitations [3, 6, 19, 32]. 

All indicators achieved perfect agreement among experts (I-CVI = 1.00), indicating a high level of consensus regarding their relevance. While this reflects strong agreement, the findings should be interpreted with caution due to the relatively small expert panel and potential homogeneity in experts’ perspectives [29, 41, 45]. 

The strong agreement observed for the dimensions of collaborative governance and organizational outcomes suggests that structural and institutional mechanisms play a critical role in enabling effective collaboration for CSA prevention. Previous studies on interprofessional collaboration in primary care have consistently emphasized the importance of leadership support, clear policies, and coordination mechanisms in facilitating teamwork across professional boundaries [8–11, 32, 45]. Governance structures help translate collaborative intentions into operational practices, ensuring that preventive activities are supported by clear procedures, accountability mechanisms, and resource allocation [19, 32, 45]. 

Similarly, the validation of the work behaviors and attitudes and collaborative commitment dimensions underscores the importance of interpersonal processes in collaborative practice. Effective communication, mutual respect, and shared responsibility are widely recognized as essential components of successful IPC [8–11, 16, 35, 50]. In the context of CSA prevention, these interpersonal dynamics are particularly important because the identification and management of child protection concerns often require coordinated responses among health professionals, social workers, and other relevant sectors [3, 17, 30, 51]. 

Further studies are required to evaluate the model’s construct validity, reliability, and practical effectiveness in primary health care settings [27, 39, 52, 53]. 

### Cultural and contextual considerations in Indonesia

Although the model includes a cultural dimension, the implementation of collaborative child protection practices must also be understood within the broader socio-cultural context of Indonesia. Cultural norms related to family privacy, social hierarchy, and community perceptions of child protection may influence how CSA cases are identified, discussed, and reported [13–15, 44]. 

In many Indonesian communities, cultural norms emphasizing family privacy and social harmony may hinder open disclosure of CSA cases, thereby affecting early prevention efforts. In many Indonesian communities, issues related to sexual abuse are often considered highly sensitive, and disclosure may be discouraged due to concerns about family reputation or social stigma [13–15, 44]. These cultural dynamics may create barriers for health professionals attempting to identify or report suspected abuse cases. Professionals working in PHC settings may therefore face complex ethical and cultural dilemmas when balancing the responsibility to protect children with respect for family and community norms [3, 51, 54]. 

The cultural context identified in this study provides important insights for the practical implementation of the model in Indonesian primary health care setting. Values such as mutual respect, collective responsibility, and community orientation can be leveraged to strengthen interprofessional collaboration in CSA prevention [8, 10, 17, 30]. In practice, the model can be implemented through culturally sensitive training programs, strengthening team-based communication, and fostering collaborative decision-making among health professionals [11, 19, 30]. 

In the Indonesian PHC context, this may include integrating local cultural values into team coordination mechanisms, community engagement initiatives, and intersectoral collaboration with social and child protection services [16, 35, 51]. These strategies highlight the importance of adapting IPC models to local cultural contexts to enhance their acceptability and effectiveness [6, 19, 32]. 

Furthermore, hierarchical professional cultures within health systems may influence communication patterns among team members. In settings where professional hierarchies are strongly embedded, open dialogue and collaborative decision-making across disciplines may require deliberate organizational support and capacity-building efforts [10, 47, 50]. 

Therefore, the successful implementation of the IPC model may require culturally sensitive strategies that include professional training, community engagement, and the development of contextually appropriate reporting and referral mechanisms [19, 32, 45]. Future implementation studies should explore how socio-cultural factors influence collaborative child protection practices in different regions of Indonesia [24, 45]. 

### Implications for primary health care practice

The content-validated IPC model provides a structured framework that can guide collaborative practice within PHC settings. By integrating individual competencies, team processes, organizational support, and governance mechanisms, the model highlights the multidimensional nature of collaboration required for effective CSA prevention [6, 8–11, 35]. 

In practical terms, the model may support PHC teams by:

1. Strengthening communication and coordination among professionals involved in child protection [8–11, 16, 51],
2. Clarifying roles and responsibilities in collaborative care [33–35, 50].
3. Supporting the development of institutional policies and referral pathways [19, 32, 45], and.
4. Promoting a shared commitment to protecting children from abuse [3, 30, 35].

However, the effectiveness of the model in improving real-world outcomes remains to be tested. Future studies should therefore evaluate whether implementing this framework leads to measurable improvements in child protection practices within PHC [27, 39, 53]. 

### Limitations

This study has several limitations. First, the model has not been evaluated for construct validity or reliability using quantitative methods such as factor analysis. Second, the expert panel was relatively small and recruited through professional networks, which may introduce selection bias and limit the diversity of perspectives. Third, the applicability of the model in real-world primary health care settings has not yet been tested. Future studies should address these limitations by involving larger and more diverse samples and conducting empirical validation [27, 53]. 

### Future research directions

Further research is needed to evaluate the empirical performance of the proposed IPC model. Future studies should focus on testing the model in operational PHC environments using both quantitative and qualitative approaches [27, 53]. 

Future studies should include pilot randomized controlled trials (RCTs), implementation research, and mixed-methods evaluations to assess feasibility, effectiveness, and sustainability of the model. Future evaluations should focus on measurable outcomes, including outcomes such as referral rates, early detection, interprofessional trust, and child safety indicators [3, 5, 8, 50]. 

Potential areas for future investigation include:

1. Assessing whether implementation of the model improves referral rates for suspected CSA cases [3, 5].
2. Examining its impact on timeliness and accuracy of child protection reporting [3, 50, 54],
3. Evaluating changes in interprofessional trust and communication among health professionals [8–11, 50], and.
4. Measuring improvements in child safety and service quality indicators [3, 4, 17].

Methodologically, future research could employ mixed-methods implementation studies, pilot intervention trials, or quasi-experimental designs to evaluate the effectiveness and feasibility of the model [19, 28, 53]. Such studies would provide valuable evidence on whether the proposed framework can translate into measurable improvements in collaborative practice and child protection outcomes within primary health care systems.

Future studies are recommended to employ mixed-methods approaches to evaluate the effectiveness and implementation of the model [53, 55, 56]. 

## Conclusion

This study developed and content-validated an interprofessional collaboration (IPC) model aimed at strengthening the prevention of child sexual abuse (CSA) in primary health care (PHC). The model comprises seven interrelated dimensions and 27 operational indicators derived from literature synthesis and expert input.

All indicators achieved an I-CVI value of 1.00, indicating strong agreement among experts and excellent content validity across all items. These findings provide preliminary evidence supporting the relevance of the proposed IPC model for CSA prevention in primary health care settings. However, further studies are required to evaluate the model’s construct validity, reliability, and real-world applicability.

The model highlights the importance of integrating individual competencies, collaborative team processes, organizational support, and culturally sensitive practices in strengthening CSA prevention. In particular, governance mechanisms and interpersonal collaboration play a central role in enabling effective and sustainable interprofessional practice.

Future research should focus on testing the implementation of the model in real-world PHC settings and evaluating its impact on measurable outcomes such as referral processes, interprofessional communication, and child safety indicators.

## Data Availability

The data supporting the findings of this study are available from the corresponding author upon reasonable request.

## Abbreviations

CSA: Child Sexual Abuse
CVI: Content Validity Index
I-CVI: Item-Level Content Validity Index
IPC: Interprofessional Collaboration
IPE: Interprofessional Education
PHC: Primary Health Care
S-CVI/Ave: Scale-Level Content Validity Index (Average Method)
S-CVI/UA: Scale-Level Content Validity Index (Universal Agreement)
SOP: Standard Operating Procedure
WHO: World Health Organization
